# Temperature-Dependent Mechanical Behaviour of Polylactic Acid Plasticised with a Bio-Based Chitin–Lecithin System

**DOI:** 10.3390/polym18182257

**Published:** 2026-09-16

**Authors:** Paul Marten, Oliver Weichold

**Affiliations:** Institute for Building Materials Research, RWTH Aachen University, Schinkelstraße 3, 52062 Aachen, Germany; marten@ibac.rwth-aachen.de

**Keywords:** polylactide, mechanical properties, annealing, bio-based, plasticisation

## Abstract

Polylactic acid (PLA) is a bio-based thermoplastic whose inherent brittleness limits mechanically demanding applications. This study investigates how a bio-based chitin–lecithin plasticiser system affects the mechanical behaviour of injection-moulded PLA as a function of additive composition, testing temperature, and annealing-induced crystallisation. Tensile and notched Charpy impact tests were performed under ambient conditions, complemented by dynamical mechanical characterisation, tensile testing at 40 and 50 °C, and DSC analysis after annealing at 130 °C for 2 h. Chitin alone increased Young’s modulus from 0.95 ± 0.08 to 1.26 ± 0.02 GPa but reduced ductility and impact strength. In contrast, ternary formulations showed increased deformability at low-to-intermediate additive contents, with fracture strain increasing from 6.87 ± 0.61% for neat PLA to 27.38 ± 3.73%. Dynamical mechanical characterisation showed only a small shift in the glass-transition temperature, while tensile deformability increased strongly as the testing temperature approached the glass-transition range. Annealing lead to crystallinities of approximately 43–45% in the ternary formulations and reduced fracture strain by 86–93%. These results indicate that the chitin–lecithin system promotes deformation of the amorphous PLA phase without a pronounced reduction in bulk glass-transition temperature, while crystallisation largely suppresses this effect.

## 1. Introduction

Polylactic acid (PLA) is a widely used bio-based thermoplastic with favourable stiffness and processability, but its inherent brittleness, low strain tolerance, and limited impact resistance restrict its use in mechanically demanding applications [1]. Plasticisation is an established approach to improve PLA ductility by increasing the mobility of the amorphous polymer phase. In particular, bio-based plasticisers have received increasing attention as a means of improving PLA flexibility while maintaining the renewable character of the material [2]. Recent work with epoxidised soybean oil, for example, has demonstrated that vegetable-oil-derived plasticisers can improve the deformability of PLA, with an elongation at break of 37.1% reported at 5 phr ESO, while also affecting its thermal and crystallisation behaviour [3]. However, plasticisation is typically accompanied by trade-offs such as reduced stiffness and strength, changes in glass-transition behaviour, and possible loss of material integrity through migration or phase separation [2,4].

In a previous study, a bio-based chitin–lecithin system was established as a melt-processable plasticiser platform for PLA [5]. While lecithin alone showed phase separation and migration under melt-processing conditions, the addition of chitin enabled more stable incorporation of lecithin into injection-moulded PLA at total additive contents of up to 24 wt% [5]. Rheological and morphological characterisation suggested that lecithin provides the primary plasticising contribution, while chitin prevents migration and facilitates its distribution within the PLA matrix [5]. However, how this heterogeneous additive system affects the mechanical response of PLA and how its plasticising contribution changes with temperature and crystallisation remain insufficiently understood.

How the chitin–lecithin system modifies the mechanical behaviour of PLA requires separate consideration. In the previously established chitin–lecithin system, the increase in deformability was achieved despite only a minor decrease in the glass-transition temperature [5]. This differs from conventional miscible PLA plasticiser systems such as PLA/PEG, for which increasing plasticiser contents are accompanied by pronounced shifts in the glass transition towards lower temperatures [6]. The mechanical response of the chitin–lecithin system may therefore not be explained solely by homogeneous bulk plasticisation through a pronounced *T*_g_ reduction, but may also depend on the heterogeneous morphology of PLA, chitin, and lecithin. Chitin has been investigated as a particulate additve in PLA composites and may increase stiffness, but its mechanical effect depends strongly on dispersion and particle–matrix adhesion [7]. Lecithin, in contrast, has been reported to improve the deformability and surface characteristics of PLA-based materials, consistent with its role as a plasticising amphiphilic additive [8]. The resulting properties should therefore depend on the balance between particulate stiffening, amorphous-phase plasticisation, and structural cohesion of the ternary blend.

Testing below but close to the glass-transition range provides a sensitive means of probing the contribution of amorphous-phase mobility, as the deformation behaviour of glassy PLA is strongly affected by the testing temperature even below *T*_g_ [9]. Annealing provides a complementary mechanistic test. The injection-moulded formulations investigated here were previously shown to be fully amorphous after injection-moulding [5]. Recent work on plasticised PLA has shown that the mechanical response after crystallisation depends not only on the crystalline fraction, but also on the constraint of the remaining amorphous phase [10]. By inducing crystallisation, annealing can therefore alter the balance between amorphous-phase plasticisation and crystalline constraint.

In this work, the mechanical behaviour of injection-moulded PLA plasticised with a bio-based chitin–lecithin system is investigated as a function of additive composition, testing temperature, and annealing-induced crystallisation. Tensile and notched Charpy impact tests are used to evaluate stiffness, strength, ductility, and energy dissipation under ambient conditions. Oscillatory shear temperature sweeps are used to characterise the glass-transition behaviour, while tensile tests at 40 and 50 °C probe the macroscopic mechanical response below the glass-transition range. Annealing at 130 °C is applied to determine how crystallisation affects the mechanical mode of action of the chitin–lecithin system. Together, these experiments are used to determine whether the enhanced deformability of the chitin–lecithin system arises primarily from a shift in bulk glass-transition behaviour or from morphology-sensitive deformation of the amorphous PLA phase, and how this distinct response changes upon crystallisation.

## 2. Materials and Methods

### 2.1. Materials

Polylactic acid (PLA, Ingeo™ 2003D, NatureWorks LLC, Minnetonka, MN, USA) was used as the polymer matrix. Prior to processing, PLA was dried at 50 °C for 2 h. Chitin powder with a degree of acetylation of 97.26% and soy lecithin were purchased from TCI Deutschland GmbH (Eschborn, Germany). The degree of acetylation of the chitin was determined by CHN elemental analysis (Vario EL Cube, Elementar Analysensysteme GmbH, Langenselbold, Germany) according to dos Santos et al. [11]. Carbon and nitrogen contents of 44.08 and 6.47 wt%, respectively, corresponding to a C/N ratio of 6.81, yielded a degree of acetylation of 97.26%. Chitin and lecithin were dried under vacuum at 40 °C before processing.

### 2.2. Specimen Preparation

**Figure 1:**
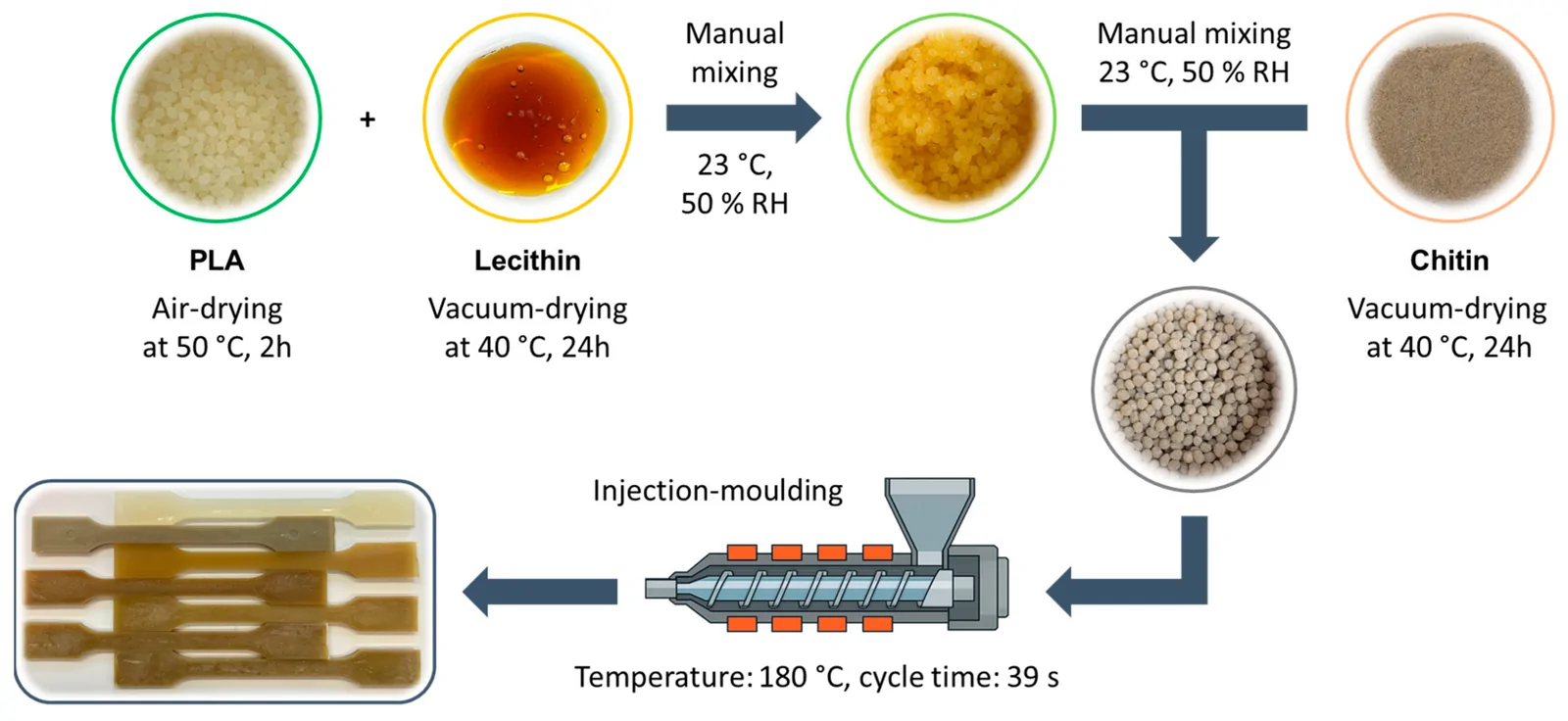
Schematic overview of the preparation of the ternary PLA–chitin–lecithin formulations.

Injection-moulded specimens were prepared using the processing route established in a previous study [5]. Briefly, PLA and lecithin were manually premixed at 23 °C and 50% relative humidity, after which chitin powder was added and incorporated manually before injection-moulding. The specimens were produced according to DIN EN ISO 3167 [12], type 1A, using an Arburg Allrounder 270A injection-moulding machine (Arburg GmbH & Co. KG, Lossburg, Germany). The barrel temperature was 180 °C, the injection pressure was 1100 bar, and the mould temperature was 30 °C. The holding time was 20 s and the total cycle time was 39 s. The processing parameters were selected based on the conditions established in our previous study [5], which provided reproducible injection-moulding of all investigated ternary formulations while maintaining sufficient thermal stability. A schematic overview of the preparation procedure is shown in Figure 1.

The investigated formulations are listed in Table 1. Neat PLA (P_100_) and PLA containing 20 wt% chitin without lecithin (P_80_C_20_) were used as reference materials. The formulations contained chitin and lecithin at a constant mass ratio of 3:1 and total additive contents between 8 and 24 wt% to assess the effect of additive loading. The 3:1 ratio was selected based on the previous study, where it represented the lowest chitin content that still provided a homogeneous and readily processable chitin–lecithin mixture and enabled successful melt processing of the ternary PLA formulations [5].

### 2.3. Mechanical Testing Under Ambient Conditions

Tensile testing under ambient conditions was carried out according to DIN EN ISO 527-1 [13] using a ZMART.PRO 1445 universal testing machine equipped with a ZwickRoell Xforce HP load cell and operated with testXpert II software, version 3.6 (ZwickRoell, Ulm, Germany). Young’s modulus was determined using a clip-on extensometer. An initial preload of 15 N was applied. The crosshead speed was 1 mm∙min^−1^ during modulus determination up to ε ≤ 0.25% and was subsequently increased to 10 mm∙min^−1^ until failure. Five specimens were tested for each formulation at 23 °C and 50% relative humidity.

Notched Charpy impact tests were performed according to DIN EN ISO 179-1 [14]. The notches were introduced using a CNC milling machine, and the tests were carried out on an in-house-built pendulum impact tester complying with ISO 13802 [15]. Five specimens were tested for each formulation at 23 °C and 50% relative humidity.

Fracture surfaces of selected specimens after notched Charpy impact testing were examined by scanning electron microscopy (SEM) using a Quattro S microscope (Thermo Fisher Scientific, Eindhoven, The Netherlands). The fracture surfaces were coated with carbon prior to analysis. Secondary electron images were recorded at 500× magnification, under high vacuum, and at an accelerating voltage of 5 kV.

### 2.4. Dynamic Mechanical Characterisation

The temperature-dependent dynamic mechanical behaviour of the formulations was investigated by oscillatory shear measurements using an Anton Paar MCR 102 rheometer (Anton Paar GmbH, Graz, Austria) equipped with a 25 mm parallel-plate geometry. Specimens were placed between the plates at 180 °C, allowed to melt, and compressed to an initial gap of 1.0 mm. The samples were then cooled from 180 to 10 °C at 5 K∙min^−1^ under normal-force control, with the normal force increasing linearly from 5 to 20 N. This resulted in a final gap of 0.500 ± 0.05 mm. Immediately after cooling, the samples were reheated from 10 to 180 °C at 5 K∙min^−1^ while maintaining a normal force of 20 N. Oscillatory measurements were performed as trifold per formulation at a strain amplitude of 0.1% and a frequency of 2 rad∙s^−1^. Data were recorded at temperature intervals of 1 °C. For graphical presentation, the curves were smoothed using a two-point moving average, whereas quantitative parameters were determined from the unsmoothed data.

### 2.5. Temperature-Dependent Tensile Testing

Temperature-dependent tensile tests were performed in an environmental chamber at 40 ± 1 °C and 50 ± 1 °C. Prior to testing, the specimens were equilibrated at the target temperature for 5 min in dry air. Because an extensometer could not be used inside the chamber, strain was calculated from crosshead displacement. The resulting Young’s moduli are therefore reported as apparent values and are used only for comparisons within this temperature-dependent test series. The crosshead speed was 1 mm∙min^−1^ in the small-strain region (ε ≤ 0.25%) and was then increased to 10 mm∙min^−1^. The maximum measurable strain was limited to 100% by the size of the chamber. Three specimens were tested for each formulation and temperature.

### 2.6. Annealing and Thermal Characterisation

Specimens were annealed isothermally at 130 °C for 2 h in a circulating-air oven and subsequently cooled to room temperature. The annealing temperature of 130 °C was selected above the cold-crystallisation range of all investigated formulations and below the PLA melting range [5]. A holding time of 2 h was used to allow extensive crystallisation before mechanical testing. Tensile tests on annealed specimens were performed at 23 °C and 50% relative humidity using the same testing protocol as for the as-moulded specimens, including clip-on extensometer measurement for Young’s modulus. Three annealed specimens were tested for each formulation.

Differential scanning calorimetry (DSC) was performed using a DSC 204 F1 Phoenix (Netzsch, Selb, Germany). Samples taken from the as-moulded and annealed specimens were analysed under a nitrogen atmosphere. Heating and cooling scans were performed between 0 and 200 °C at a rate of 10 K∙min^−1^. The glass-transition temperature was determined from the midpoint of the heat-capacity step, while the melting temperature was taken from the maximum of the melting endotherm. The degree of crystallinity of the annealed specimens was determined from the melting enthalpy obtained during the first heating scan according to Equation (1). One DSC measurement was performed for each formulation and condition.

(1) $$X_{c}=\frac{\Delta H_{m}}{w_{PLA}\cdot \Delta H_{m}^{0}}\times 100\%$$

$\Delta H_{m}$: Measured melting enthalpy;

$w_{PLA}$: Mass fraction of PLA in the formulation;

$\Delta H_{m}^{0}$: Melting enthalpy of fully crystalline PLA (93.1 J∙g^−1^) [16].

## 3. Results and Discussion

### 3.1. Mechanical Properties at Ambient Conditions

**Figure 2:**
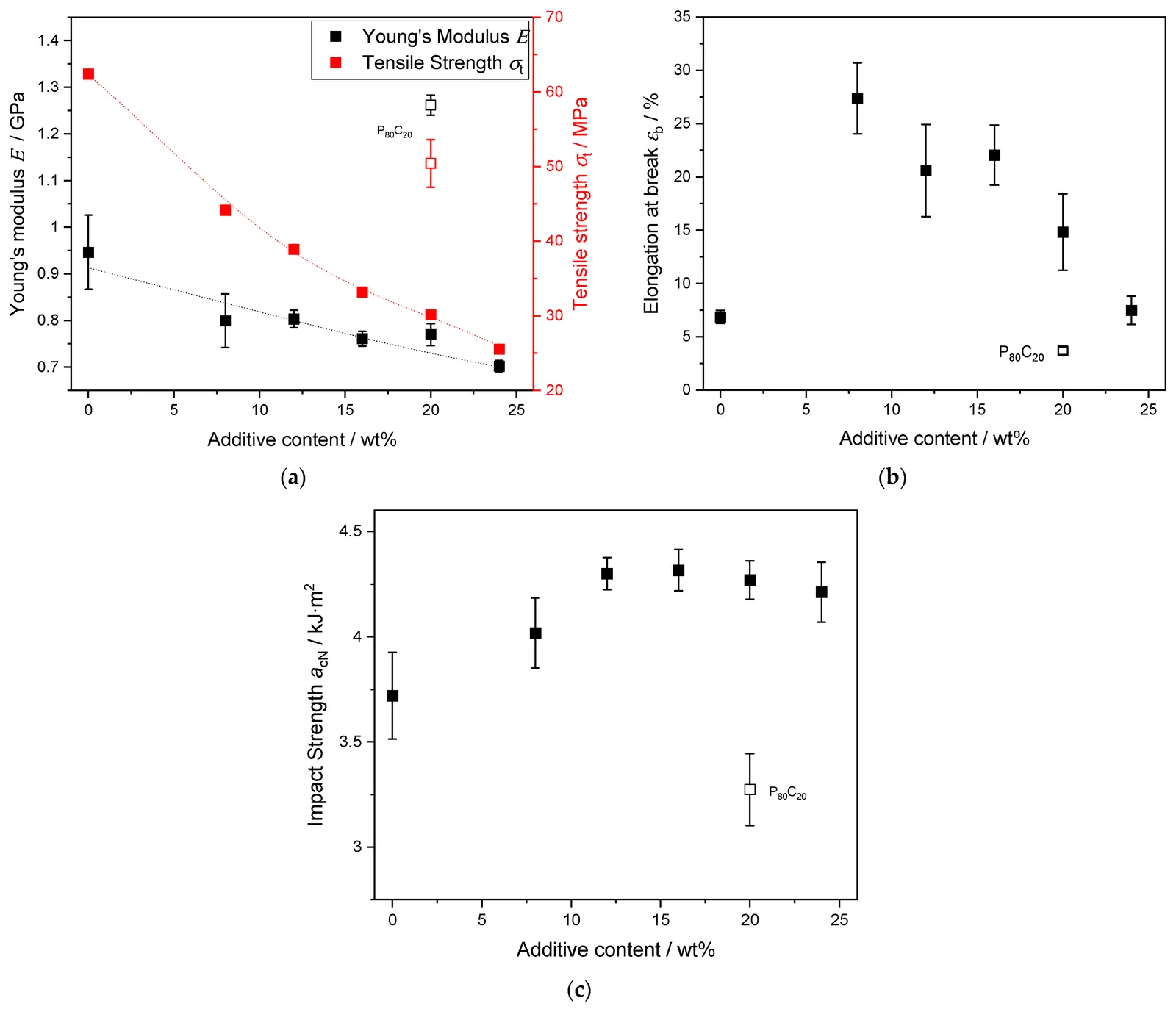
Mechanical properties of neat PLA, the chitin-only reference, and the ternary PLA–chitin–lecithin formulations under ambient conditions. (**a**) Young’s modulus and tensile strength, (**b**) elongation at break, and (**c**) notched Charpy impact strength as a function of total additive content. Error bars represent standard deviations.

The mechanical response of the injection-moulded specimens was first investigated under ambient conditions using tensile and notched Charpy impact tests. The Young’s modulus and tensile strength are shown in Figure 2a, while the elongation at break and notched Charpy impact strength are presented in Figure 2b and Figure 2c, respectively. Representative stress–strain curves and the complete tensile dataset are provided in the Supplementary Materials (Figure S1 and Table S1).

The chitin-only reference P_80_C_20_ exhibits a higher Young’s modulus than neat PLA, increasing from 0.95 ± 0.08 GPa for P_100_ to 1.26 ± 0.02 GPa. At the same time, the tensile strength decreases from 62.40 ± 0.61 MPa to 50.42 ± 0.82 MPa. The elongation at break is also reduced from 6.9 ± 0.6% for neat PLA to 3.7 ± 0.4% for P_80_C_20_ (Figure 2a,b). These results indicate that chitin increases stiffness but does not improve the resistance of PLA against tensile failure, consistent with the behaviour of a rigid particulate filler with limited matrix adhesion.

In contrast, the ternary PLA–chitin–lecithin formulations exhibit a steady decrease in Young’s modulus and tensile strength with increasing additive content (Figure 2a). In contrast, the effect on elongation at break is more pronounced and non-monotonic. The formulation P_92_C_6_L_2_, containing only 2 wt% lecithin, reaches an elongation at break of 27.4 ± 3.4%, approximately four times the value of neat PLA. Comparable values are retained up to intermediate additive contents. At the highest investigated additive loading, however, the elongation at break decreases again to 7.5 ± 1.3% for P_76_C_18_L_6_ (Figure 2b).

The pronounced increase in elongation at break at low and intermediate lecithin contents indicates that lecithin acts as the primary plasticising component within the ternary formulations. The decrease observed at the highest loading suggests that the beneficial plasticising contribution is increasingly counteracted by the high particulate content and by a loss of structural cohesion. The mechanical response therefore cannot be described by a simple monotonic plasticisation effect.

The notched Charpy impact results support this interpretation (Figure 2c). Neat PLA exhibits an impact strength of 3.72 ± 0.21 kJ∙m^−2^. The addition of chitin alone reduces the impact strength to 3.27 ± 0.17 kJ∙m^−2^ for P_80_C_20_, consistent with the lower tensile ductility of this formulation. In contrast, the ternary blends exhibit increased impact resistance, reaching 4.32 ± 0.10 kJ∙m^−2^ for P_84_C_12_L_4_. Further increasing the additive content does not result in a corresponding additional improvement, and the impact strength approaches a plateau within the experimental uncertainty.

**Figure 3:**
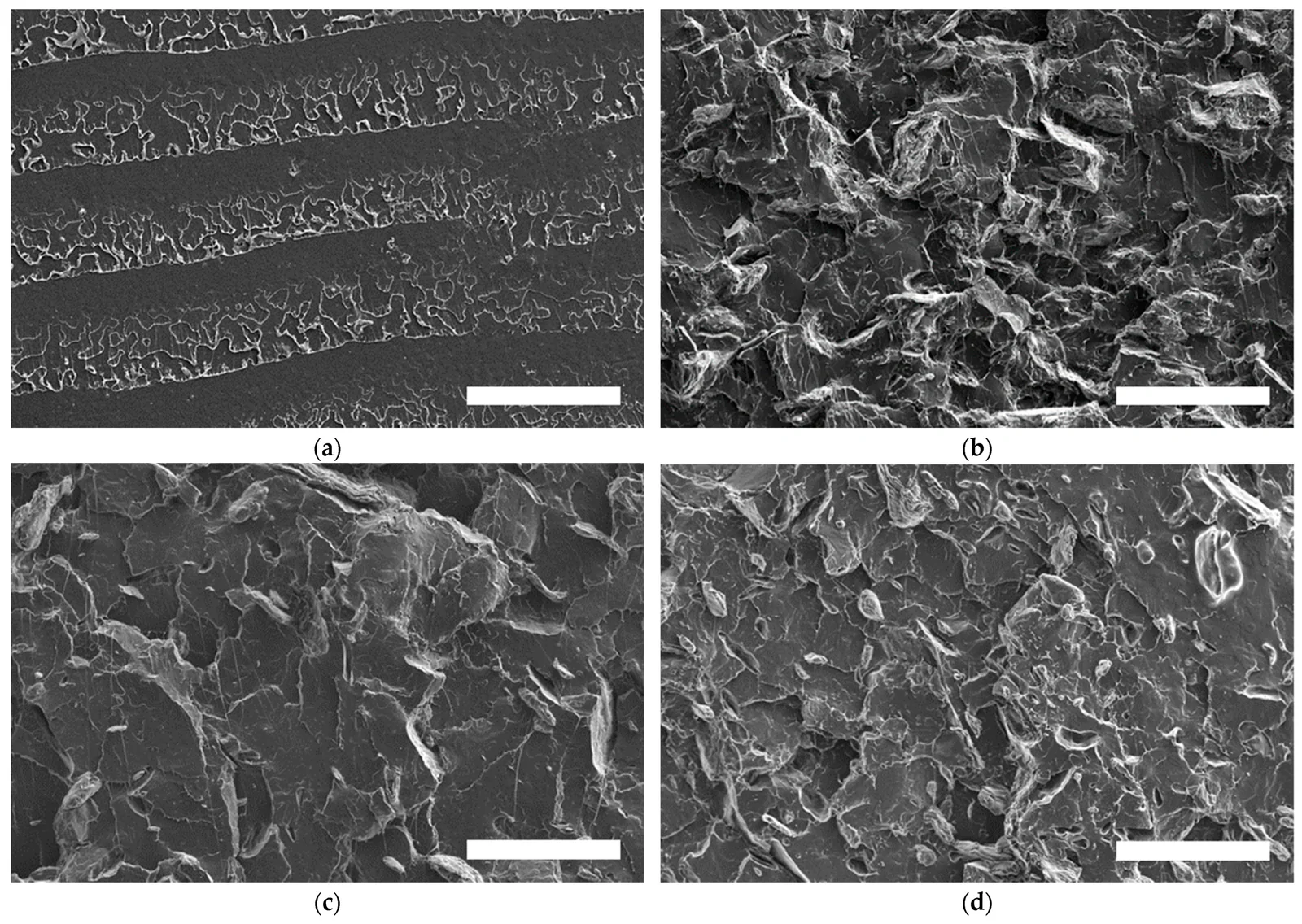
SEM micrographs of fracture surfaces obtained after notched Charpy impact testing at 500× magnification: (**a**) neat PLA (P_100_), (**b**) P_80_C_20_, (**c**) P_92_C_6_L_2_, and (**d**) P_80_C_15_L_5_. Scale bars: 100 µm.

SEM micrographs of the fracture surfaces after notched Charpy impact testing are shown in Figure 3. Neat PLA exhibits comparatively smooth regions with river-like markings, consistent with rapid crack propagation and limited plastic deformation. P_80_C_20_ shows a much rougher and more fragmented surface. Since its impact strength is lower than that of neat PLA, this roughness is more likely associated with crack deflection and local interfacial failure than with effective energy dissipation, consistent with the interfacial gaps observed previously [5]. In contrast, P_92_C_6_L_2_ and P_80_C_15_L_5_ show more continuous matrix regions with irregular tearing and deformation features, indicating a greater contribution of matrix deformation during fracture. The direct comparison between P_80_C_20_ and P_80_C_15_L_5_ at the same total additive content further suggests that lecithin changes the failure behaviour rather than merely increasing structural heterogeneity.

Taken together, the tensile and impact results reveal two competing contributions. Chitin alone predominantly increases stiffness, whereas lecithin promotes deformation and energy dissipation. The marked increase in fracture strain at low lecithin content, despite the simultaneous decrease in stiffness and tensile strength, reflects the expected trade-off associated with plasticisation. The most favourable balance is obtained at low-to-intermediate additive contents. At higher loadings, the increasing particulate fraction appears to counteract the beneficial plasticising contribution, limiting further improvements in ductility and impact resistance. For comparison, P_92_C_6_L_2_ reached an elongation at break of 27.4% at only 2 wt% lecithin, which is comparable to values reported for PLA plasticised with other bio-based systems like epoxidised soybean oil [3].

### 3.2. Dynamic Mechanical Characterisation

**Figure 4:**
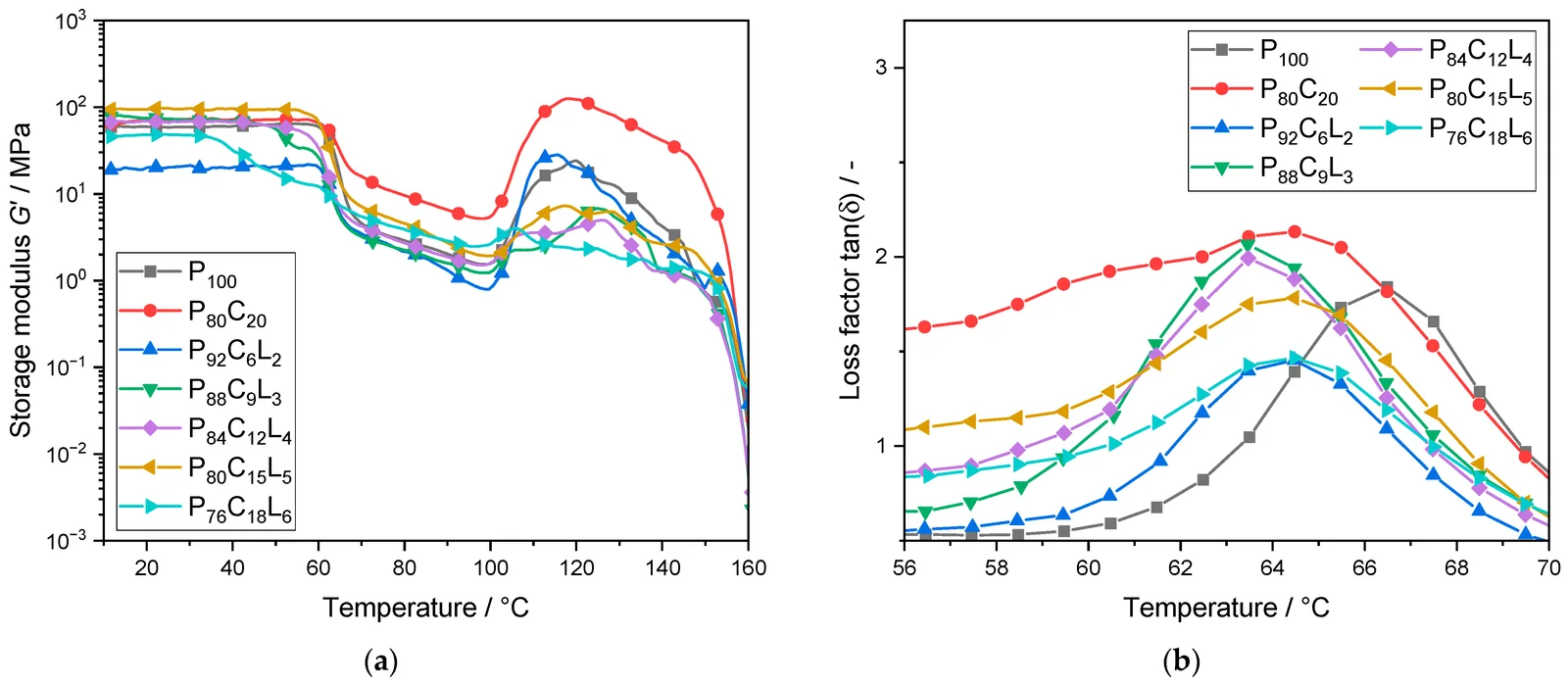
Temperature-dependent oscillatory shear behaviour of neat PLA (P_100_), the chitin-only reference (P_80_C_20_), and the ternary PLA–chitin–lecithin formulations. (**a**) Storage modulus *G*′ during heating from 10 to 160 °C and (**b**) loss factor tan(*δ*) in the glass-transition region. All specimens were subjected to an identical melt–cooling cycle prior to heating.

To further examine the temperature-dependent mechanical response of the formulations in the vicinity of the glass transition, oscillatory shear temperature sweeps were performed. The temperature dependence of the storage modulus (*G*′) over the complete heating range and the loss factor (tan(*δ*)) in the glass-transition region are shown in Figure 4, while the corresponding loss modulus (*G*″) and the loss factor over the complete heating range from 10 to 180 °C are provided in the Supplementary Materials (Figures S2 and S3, respectively).

At temperatures below the glass-transition region, most formulations exhibited a comparatively stable storage modulus. Upon heating, *G*′ decreased markedly in the transition region, reflecting the increasing segmental mobility of the PLA phase. The formulation with the highest additive content, P_76_C_18_L_6_, additionally exhibited a decrease in both *G*′ and *G*″ at lower temperatures, below the glass-transition region. Since this behaviour was not accompanied by a distinct additional maximum in tan(*δ*), it is interpreted as a broad pre-transition softening rather than as evidence of a separate relaxation process.

The glass-transition temperature was evaluated from the maximum of the loss factor tan(δ). The corresponding values are summarised in Table 2.

Neat PLA exhibited the highest glass-transition temperature, with the maximum occurring at 66 ± 2 °C, whereas the chitin-only reference P_80_C_20_ and the ternary PLA-chitin–lecithin formulations showed values of approximately 63–64 °C. The corresponding DSC values reported previously show a similar limited decrease in *T*_g_ from 59.4 °C for neat PLA to approximately 57 to 59 °C for the modified formulations. The systematically higher transition temperatures obtained from oscillatory shear are attributed to the different experimental methods and heating rates. Thus, both methods indicate that incorporation of the chitin–lecithin system causes only a minor shift in the bulk glass-transition temperature.

The limited shift in the tan(*δ*)-maximum is particularly notable in view of the pronounced differences in tensile deformability observed between the formulations. The enhanced deformation of the ternary blends therefore does not coincide with a correspondingly large shift in the glass-transition region. This differs from miscible PLA plasticiser systems such as PLA/PEG, for which increasing plasticiser contents have been reported to produce substantially larger shifts in the tan(*δ*)-maximum towards lower temperatures [6]. The present results are therefore consistent with the interpretation that the mechanical effect of the chitin–lecithin system cannot be described solely by homogeneous bulk plasticisation through a pronounced reduction in *T*_g_. Instead, the response may depend on the combined effects of increased PLA-chain mobility and the heterogeneous morphology of the ternary system.

Above the glass-transition region, the storage modulus *G*′ increased again before decreasing at higher temperatures. This modulus recovery is attributed to cold crystallisation of PLA during heating. The increase was particularly pronounced for the chitin-only reference P_80_C_20_ and became less pronounced with increasing chitin–lecithin content. This behaviour is consistent with the previously observed reduction in cold crystallisation with increasing chitin–lecithin content [5]. At higher temperatures (>150 °C), tan(*δ*) increased strongly as the PLA melting range was approached, reflecting the loss of elastic character as *G*′ decreased.

### 3.3. Temperature-Dependent Tensile Behaviour

To assess the influence of temperature on the mechanical response, tensile tests were performed at 40 and 50 °C. Both temperatures remain below the glass-transition range of the investigated formulations, which is approximately 57–59 °C as determined by DSC in a previous study [5]. Because an extensometer could not be used inside the environmental chamber, strain was derived from crosshead displacement. Since crosshead displacement also includes compliance of the testing system, the absolute strain values should be interpreted with caution and are primarily used for comparison between formulations tested under identical conditions. The resulting Young’s moduli are therefore reported as apparent values and are used for within-series comparisons only.

**Figure 5:**
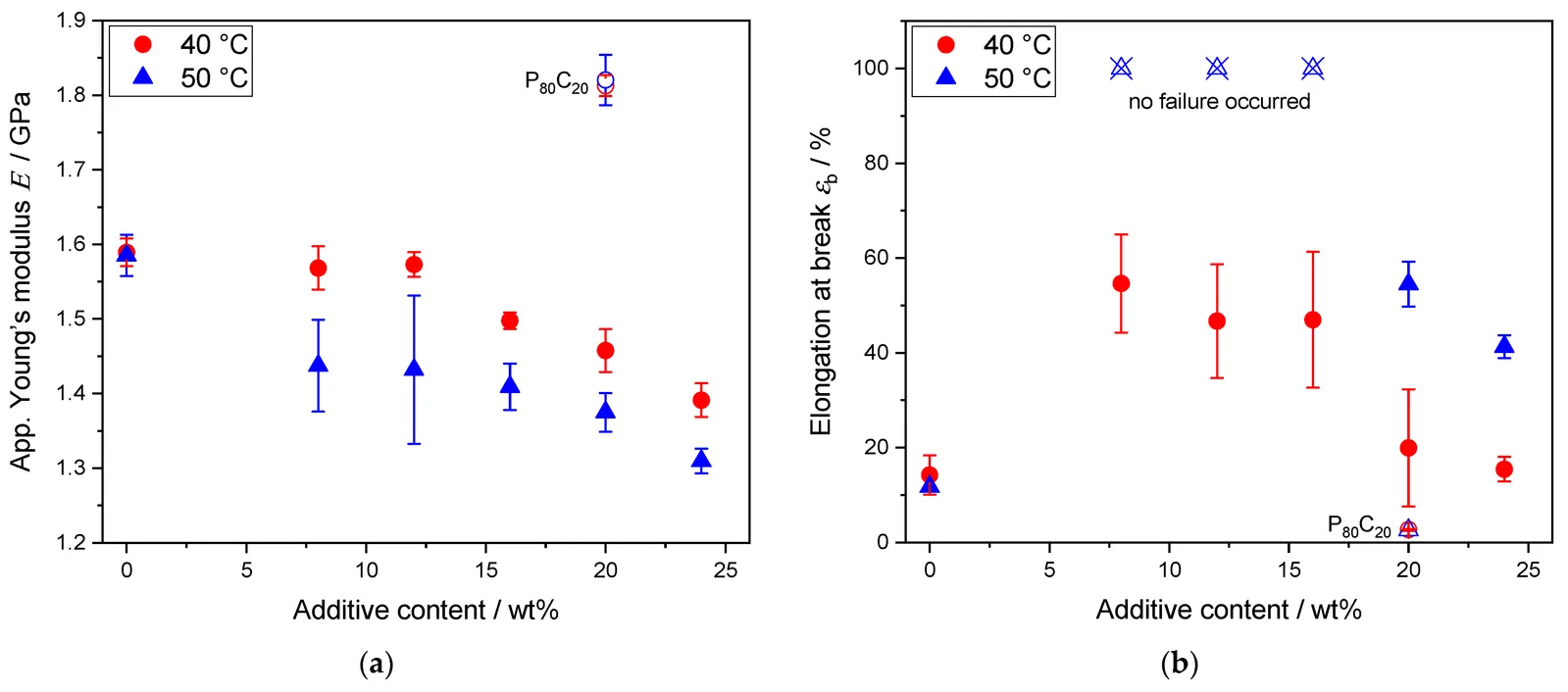
(**a**) Apparent Young’s modulus and (**b**) elongation at break as a function of the additive content at 40 °C and 50 °C. No failure occurred within the travel limits (100% elongation) of the temperature chamber for the specimens with 8, 12 and 16 wt% additive content (crossed-out symbols). The open symbols represent the chitin-only reference P_80_C_20_.

The apparent Young’s modulus and the strain at failure are shown in Figure 5a and Figure 5b, respectively. Neat PLA (P_100_) and the chitin-only reference (P_80_C_20_) retain a comparatively brittle response at both temperatures. In contrast, the ternary PLA–chitin–lecithin formulations exhibit substantially increased deformability. For several formulations containing low-to-intermediate additive contents, no failure occurred within the maximum measurable strain of 100%. The corresponding values therefore represent lower limits rather than measured elongations at break.

The difference between the reference specimens and the ternary formulations becomes more pronounced at 50 °C. As the testing temperature approaches the glass-transition range, the apparent Young’s modulus decreases and the strain tolerance of the lecithin-containing blends increases markedly. This pronounced change is notable because the glass-transition temperatures of the formulations differ only slightly. The results indicate that the mechanical response below *T*_g_ is highly sensitive to the plasticising contribution of lecithin, even though the shift in the bulk glass transition remains limited.

The comparison with P_80_C_20_ shows that the effect cannot be attributed solely to the presence of chitin particles. Although P_80_C_20_ contains a high particulate fraction, it does not exhibit a comparable increase in deformability. Chitin alone predominantly contributes to stiffness, whereas the presence of lecithin promotes deformation of the PLA matrix. The temperature-dependent behaviour is therefore consistent with a plasticisation-dominated response of the ternary formulations.

**Figure 6:**
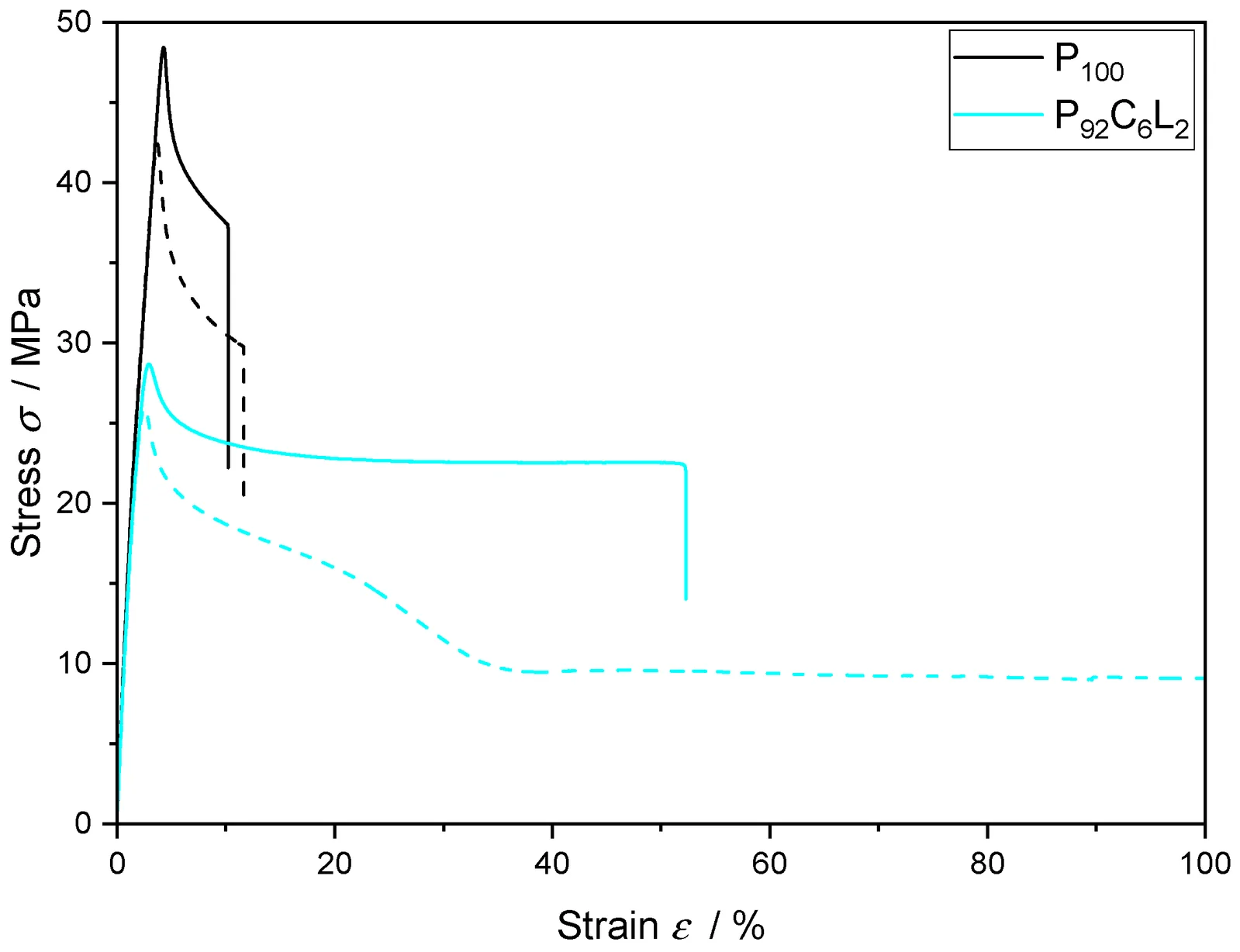
Representative stress–strain curves of neat PLA (P_100_) and P_92_C_6_L_2_ measured at 40 °C (solid line) and 50 °C (dotted line). Strain was derived from crosshead displacement.

Representative stress–strain curves of neat PLA (P_100_) and P_92_C_6_L_2_ measured at 40 and 50 °C are shown in Figure 6. While neat PLA fails at comparatively low strain, the lecithin-containing formulations exhibit pronounced deformation, particularly at 50 °C. Tensile strengths measured at both temperatures are provided in the Supplementary Materials (Figure S4). The decrease in tensile strength with increasing temperature observed for all formulations is consistent with progressive softening as the glass-transition range is approached. However, the change is less pronounced than the change in the apparent Young’s modulus and the elongation at break.

Taken together, the temperature-dependent tensile results reveal that the mechanical response of the ternary formulations is strongly influenced by the state of the amorphous PLA phase. The plasticising contribution of lecithin is already detectable under ambient conditions but becomes substantially more pronounced as the testing temperature approaches *T*_g_. The pronounced increase in deformability despite only a minor shift in bulk glass-transition temperature suggests that the chitin–lecithin system does not act primarily through homogeneous bulk plasticisation. Instead, the mechanical response appears to involve morphology-sensitive enhancement of deformation within the amorphous PLA phase. Whether this enhanced deformability is retained after the development of a crystalline morphology is examined in the following section.

### 3.4. Influence of Annealing on Crystalline Morphology and Mechanical Behaviour

To investigate how the mechanical response changes upon development of a semi-crystalline morphology, the injection-moulded specimens were annealed at 130 °C for 2 h. This temperature was selected above the cold-crystallisation range of all investigated formulations. The annealed specimens were subsequently analysed by DSC and tensile testing in order to assess how crystallisation affects the balance between plasticisation of the amorphous PLA phase and the stiffening contribution of the crystalline morphology.

**Figure 7:**
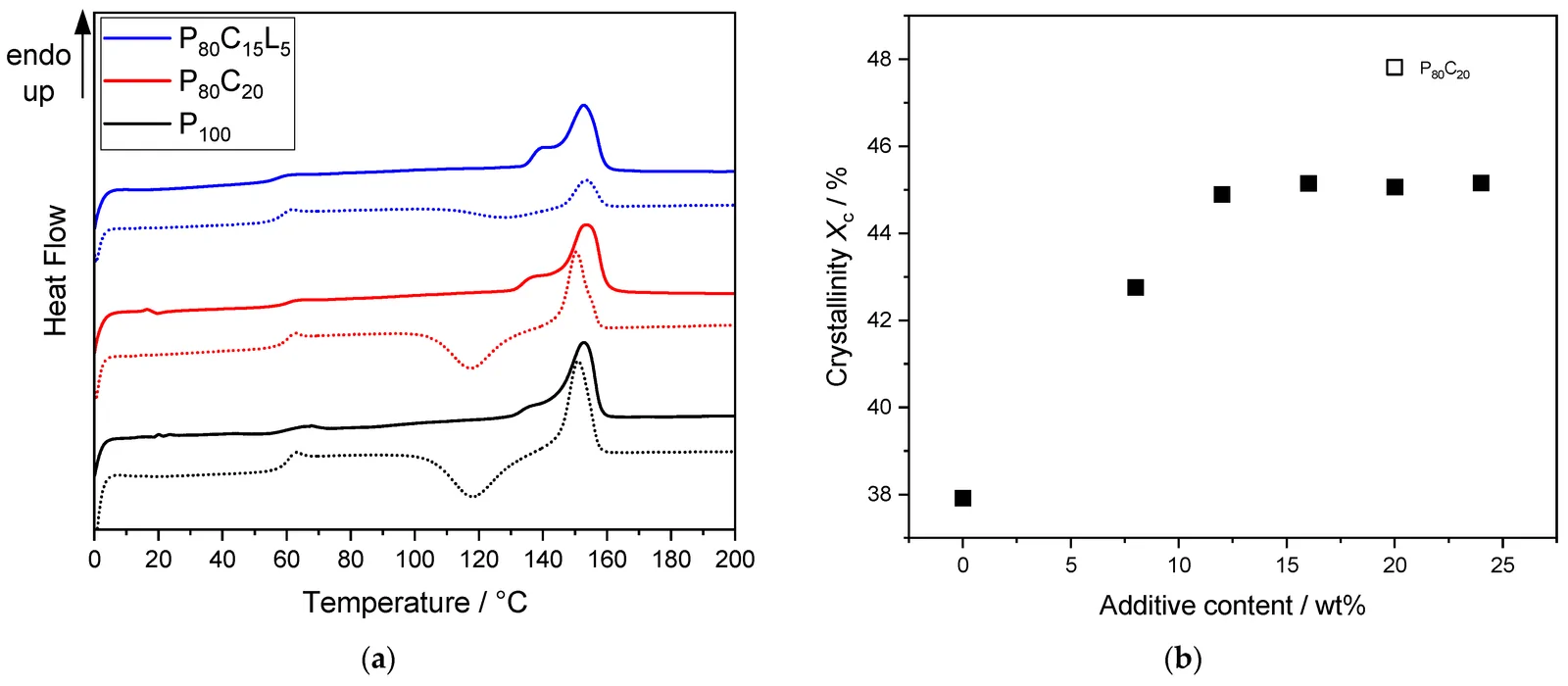
(**a**) Comparison between the first-heating DSC curves before (dotted line) and after (solid line) annealing for neat PLA (P_100_), the chitin-only reference (P_80_C_20_), and a representative blend (P_80_C_15_L_5_). (**b**) Degree of crystallinity after annealing, *X*_c_, as a function of total additive content. The open symbol represents the chitin-only reference P_80_C_20_. The degree of crystallinity was corrected for the PLA fraction.

The first-heating DSC curves of the annealed specimens no longer exhibit a cold-crystallisation exotherm, indicating that crystallisation occurred during the thermal treatment (Figure 7a). The degree of crystallinity is in the range of 38% for neat PLA to 43–45% for the ternary PLA–chitin–lecithin formulations (Figure 7b). Within the ternary series, the crystallinity approaches a plateau at intermediate additive contents and remains close to 45% upon further increasing the chitin–lecithin content. The chitin-only reference without lecithin P_80_C_20_ exhibits the highest degree of crystallinity, reaching approximately 48%.

The higher crystallinity of P_80_C_20_ compared to neat PLA subjected to the same annealing treatment suggests that chitin can promote crystallisation during annealing, potentially by providing heterogeneous nucleation sites. However, the crystallinity does not increase further when lecithin is introduced into the formulations. Instead, the ternary blends approach a comparatively narrow plateau. While chitin may facilitate nucleation, lecithin may interfere with regular PLA chain packing, thereby limiting the extent of crystallisation during annealing. The final crystalline morphology of the ternary blends may therefore reflect a balance between a possible nucleating contribution of chitin and a packing-disrupting influence of lecithin.

The annealed specimens additionally exhibit a split melting endotherm. Multiple melting behaviour is frequently observed for PLA and is commonly associated with crystal populations of different perfection and with melting–recrystallisation processes during heating [17]. The present DSC data do not allow an unambiguous assignment of the individual melting contributions to specific crystal modifications. Nevertheless, the appearance of multiple melting contributions confirms that annealing substantially modifies the crystalline morphology compared to the predominantly amorphous as-moulded state.

**Figure 8:**
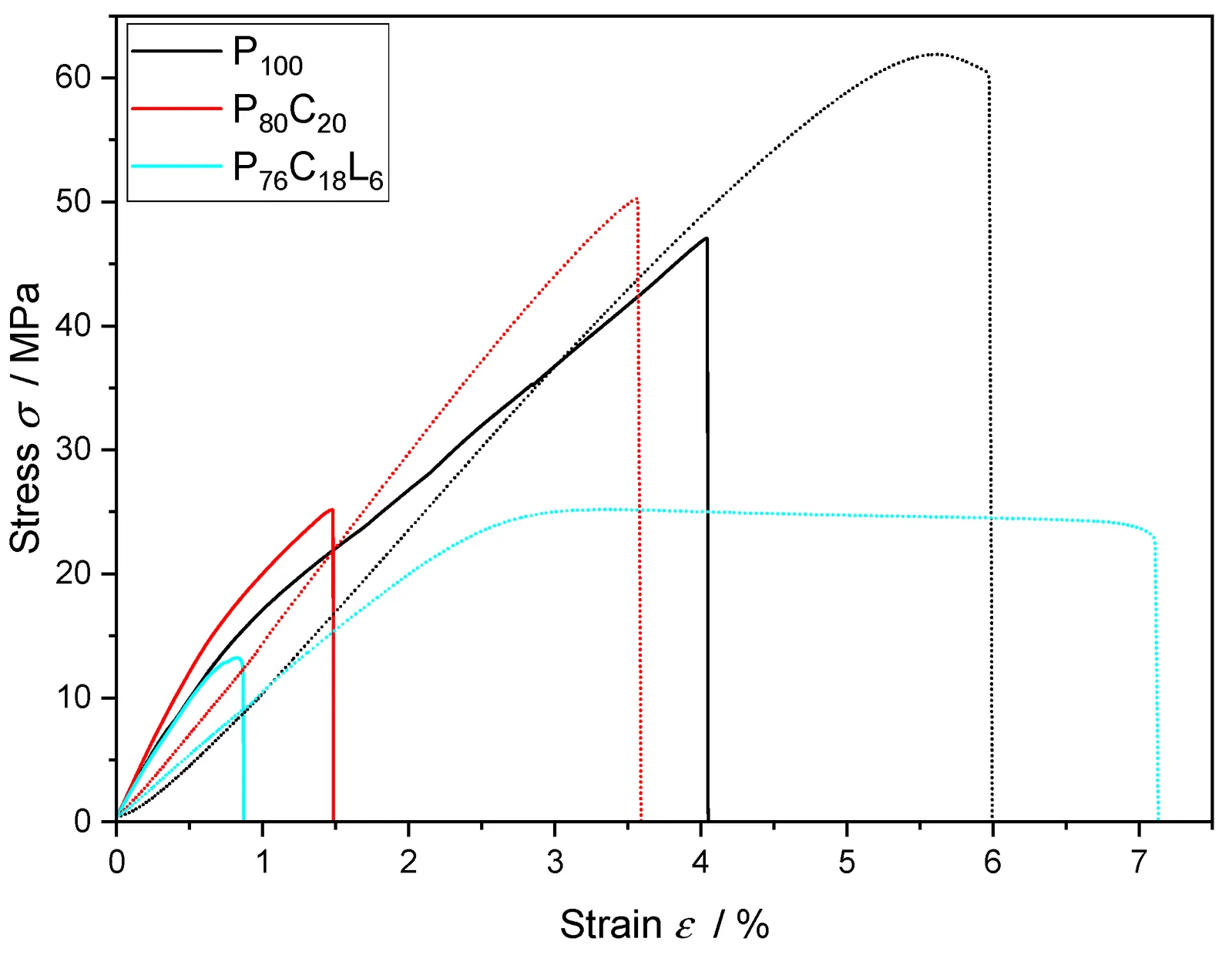
Representative comparison between the stress–strain curves before (dotted line) and after (solid line) annealing at 130 °C for 2 h of the neat PLA (P_100_), the chitin-only reference (P_80_C_20_), and a representative blend (P_80_C_15_L_5_).

The development of the semi-crystalline morphology strongly affects the tensile behaviour (Figure 8). After annealing, the Young’s modulus increases substantially for all formulations, while tensile strength and elongation at break decrease. Representative stress–strain curves before and after annealing are shown in Figure 8. The absolute tensile properties of the annealed specimens are summarised in the Supplementary Materials (Table S3), while the corresponding relative changes are provided in Table S4.

Neat PLA and the chitin-only reference exhibit relative increases in Young’s modulus of approximately 390 and 370%, respectively. The ternary PLA–chitin–lecithin formulations show stronger increases ranging from approximately 455 to 578%. At the same time, tensile strength decreases by approximately 22–47% for the ternary blends, while the elongation at break is reduced by approximately 86–93%. The reduction in elongation at break is therefore particularly pronounced for the lecithin-containing formulations.

The simultaneous increase in stiffness and decrease in tensile strength and elongation at break show that annealing does not simply reinforce the material. Instead, the crystalline regions constrain deformation of the amorphous phase and reduce the ability of the specimens to redistribute local stresses before failure. In the as-moulded state, the plasticising contribution of lecithin promotes deformation of the PLA matrix. After annealing, this contribution is largely overridden by the semi-crystalline morphology established during thermal treatment.

This interpretation is consistent with recent work on plasticised PLA showing that the mechanical response of semi-crystalline PLA is governed not only by the crystalline fraction, but also by the constrained amorphous interphase between mobile amorphous regions and crystalline domains [10]. During crystallisation, lecithin is unlikely to be incorporated into the PLA crystals and may therefore become enriched in the remaining amorphous phase. Such enrichment could locally increase the plasticising contribution of lecithin. However, the pronounced reduction in elongation at break after annealing indicates that any such effect is insufficient to compensate for the restriction of molecular deformation imposed by the semi-crystalline morphology.

The comparison between the as-moulded and annealed states therefore reveals a competition between two structural contributions. Plasticisation of the amorphous phase dominates the deformability of the as-moulded ternary blends, whereas crystallisation increasingly governs the mechanical response after annealing. The final properties of the chitin–lecithin-modified PLA specimens are thus determined not only by additive composition, but also by the thermal history and the crystalline morphology established during processing or post-treatment.

## 4. Conclusions

This study shows that the mechanical response of injection-moulded PLA modified with a bio-based chitin–lecithin system reflects a balance between particulate stiffening, amorphous-phase plasticisation, and morphology. Chitin alone increases stiffness but does not improve ductility or impact resistance, whereas lecithin promotes deformation and energy dissipation in the ternary formulations. The most favourable mechanical response is therefore obtained at low-to-intermediate chitin–lecithin contents, where the plasticising contribution is not yet outweighed by the increasing particulate fraction.

Oscillatory shear measurements showed only a small shift in the glass-transition temperature determined from the maximum of tan(*δ*) between the formulations, indicating that the pronounced differences in deformability are not accompanied by a correspondingly large shift in the glass-transition temperature. Temperature-dependent tensile testing showed that the enhanced deformability of the ternary formulations becomes more pronounced as the testing temperature approaches *T*_g_, consistent with an increasing contribution of amorphous-phase mobility. Conversely, annealing-induced crystallisation largely suppresses the gained ductility by constraining the amorphous phase. The chitin–lecithin system should therefore be understood as a morphology-sensitive plasticising approach whose effectiveness depends on additive composition, testing temperature, and thermal history.

Among the investigated formulations, P_92_C_6_L_2_ provided the highest tensile deformability, whereas P_84_C_12_L_4_ exhibited the highest notched Charpy impact strength. The preferred formulation therefore depends on the required balance between stiffness, strength, ductility, and impact resistance. Such formulations may be particularly relevant for injection-moulded components such as clips, housings, or covers, where increased ductility and impact resistance are required at moderate service temperatures.

## Figures and Tables

**Table 1:** Composition of the injection-moulded specimens.

Specimen	Composition [wt%]		
	PLA	Chitin	Lecithin
P_100_	100	0	0
P_80_C_20_	80	20	0
P_92_C_6_L_2_	92	6	2
P_88_C_9_L_3_	88	9	3
P_84_C_12_L_4_	84	12	4
P_80_C_15_L_5_	80	15	5
P_76_C_18_L_6_	76	18	6

**Table 2:** Glass-transition temperatures determined from the maximum of the loss factor tan(*δ*) during oscillatory shear temperature sweeps and corresponding DSC values reported previously [5]. Oscillatory shear values are reported as mean ± standard deviation (*n* = 3).

Specimen	Composition [wt%]			*T*_g,tan(*δ*)_ [°C]	*T*_g,DSC_ [°C] ^a^
	PLA	Chitin	Lecithin		
P_100_	100	0	0	66 ± 2	59.4
P_80_C_20_	80	20	0	64 ± 1	59.1
P_92_C_6_L_2_	92	6	2	63 ± 1	57.6
P_88_C_9_L_3_	88	9	3	63 ± 1	57.4
P_84_C_12_L_4_	84	12	4	64 ± 1	57.0
P_80_C_15_L_5_	80	15	5	64 ± 1	57.2
P_76_C_18_L_6_	76	18	6	64 ± 1	57.0

^a^ Values reported previously in ref. [5].

## Data Availability

The original contributions presented in this study are included in the article/Supplementary Materials. Further inquiries can be directed to the corresponding authors.

## Supplementary Materials

The following supporting information can be downloaded at https://www.mdpi.com/article/10.3390/polym18182257/s1, Table S1: Tensile properties of the specimens containing PLA, Chitin and Soy lecithin at RT; Figure S1: Representative stress–strain curves of the specimens under ambient conditions; Figure S2: Temperature dependence of the loss modulus *G*″ of the specimens during oscillatory shear heating measurements from 10 to 160 °C; Figure S3: Temperature dependence of the loss factor tan δ of the specimens during oscillatory shear heating measurements. The maximum in tan(*δ*) around 63–66 °C corresponds to the glass-transition region, while the pronounced increase above approximately 150 °C coincides with the PLA melting range determined by DSC [5]; Figure S4: Tensile strength as a function of total additive content at 40 and 50 °C; Table S2: Thermal properties of the specimens after annealing at 130 °C for 2 h; Table S3: Tensile properties of the specimens after annealing at 130 °C for 2 h; Table S4: Relative changes in tensile properties after annealing at 130 °C for 2 h. The relative changes were calculated as Δ*X* = (*X*_annealed_ − *X*_as-moulded_)/*X*_as-moulded_ · 100%.

## Abbreviations

The following abbreviations are used in this manuscript:

PLA	Polylactic acid
DSC	Differential scanning calorimetry
CNC	Computer numerical control
RT	Room temperature
