# Supplementary material for: Temperature-Dependent Mechanical Behaviour of Polylactic Acid Plasticised with a Bio-Based Chitin–Lecithin System
Source: Polymers (Basel). 2026 Sep 16;18(18):2257. doi: 10.3390/polym18182257 (PMC13611398; doi:10.3390/polym18182257)
Supplement: Supplementary file 1 [file polymers-18-02257-s001.zip › polymers-4558678-supplementary.pdf]

Supplementary materials

Temperature-Dependent Mechanical Behaviour of Polylactic Acid Plasticised with a Bio-Based Chitin-Lecithin System

Paul Marten and Oliver Weichold*

RWTH Aachen University, Institute for Building Materials Research, Schinkelstraße 3, 52062 Aachen, Germany

* Author to whom correspondence should be addressed: weichold@ibac.rwth-aachen.de

Table S1: Tensile properties of the specimens containing PLA, Chitin and Soy lecithin at RT.

Specimen	Young's modulus *E* [GPa]	Tensile strength σ_y [MPa]	Strain at tensile strength ε_y [%]	Fracture stress σ_b [MPa]	Fracture strain ε_b [%]
P_{100}	0.946 ± 0.079	62.40 ± 0.61	5.75 ± 0.09	58.46 ± 1.46	6.87 ± 0.61
$P_{80}C_{20}$	1.26 ± 0.02	50.42 ± 0.82	3.67 ± 0.35	49.22 ± 3.50	3.68 ± 0.35
$P_{92}C_6L_2$	0.800 ± 0.058	44.16 ± 0.22	4.73 ± 0.19	37.11 ± 0.44	27.38 ± 3.73
$P_{88}C_9L_3$	0.803 ± 0.019	38.92 ± 0.13	3.97 ± 0.08	32.31 ± 0.64	20.56 ± 4.84
$P_{84}C_{12}L_4$	0.761 ± 0.016	33.18 ± 0.29	3.73 ± 0.15	28.51 ± 0.84	22.25 ± 2.77
$P_{80}C_{15}L_5$	0.770 ± 0.023	30.15 ± 0.17	3.36 ± 0.06	25.14 ± 0.45	14.83 ± 3.59
$P_{76}C_{18}L_6$	0.702 ± 0.012	25.54 ± 0.57	3.28 ± 0.15	22.21 ± 0.87	7.49 ± 1.33

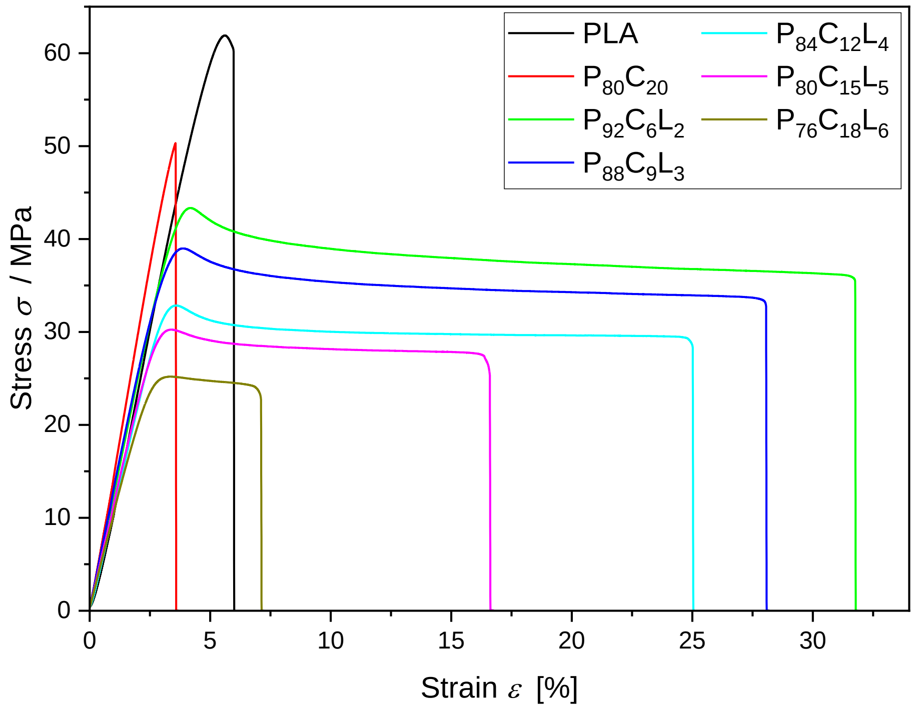

Figure S1: Representative stress-strain curves of the specimens under ambient conditions.

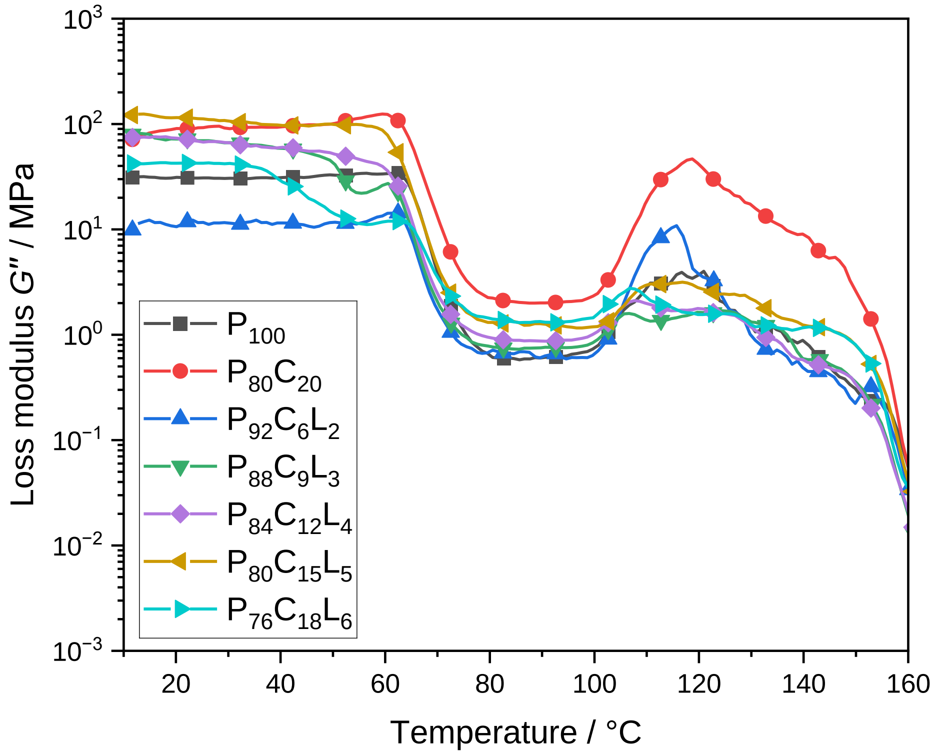

Figure S2: Temperature dependence of the loss modulus *G*″ of the specimens during oscillatory shear heating measurements from 10 to 160 °C.

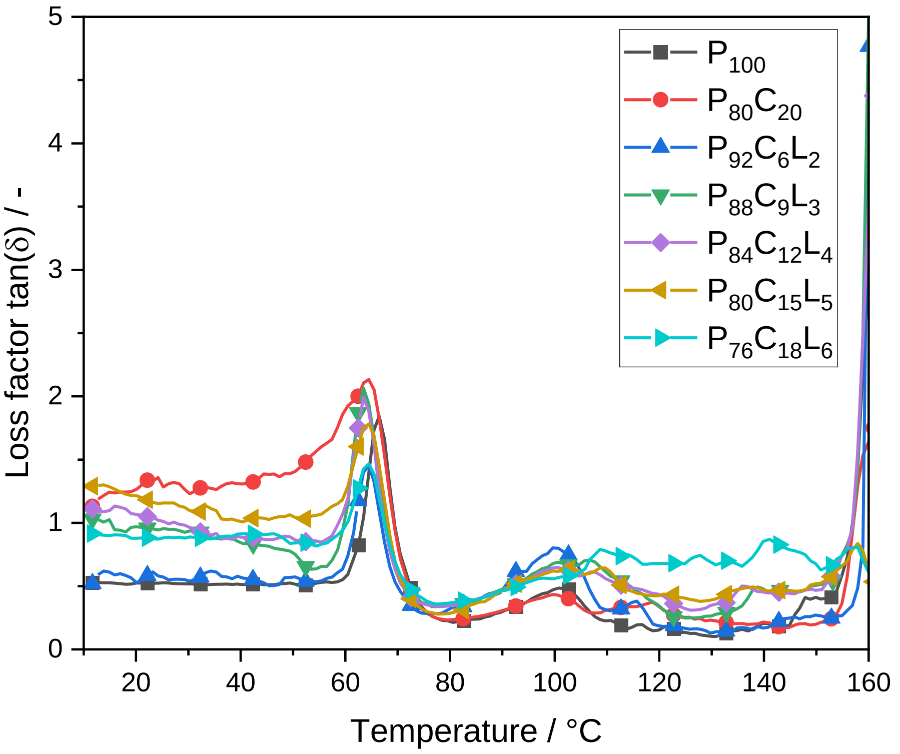

Figure S3: Temperature dependence of the loss factor tan δ of the specimens during oscillatory shear heating measurements. The maximum in tan(δ) around 63–66 °C corresponds to the glass-transition region, while the pronounced increase above approximately 150 °C coincides with the PLA melting range determined by DSC [5].

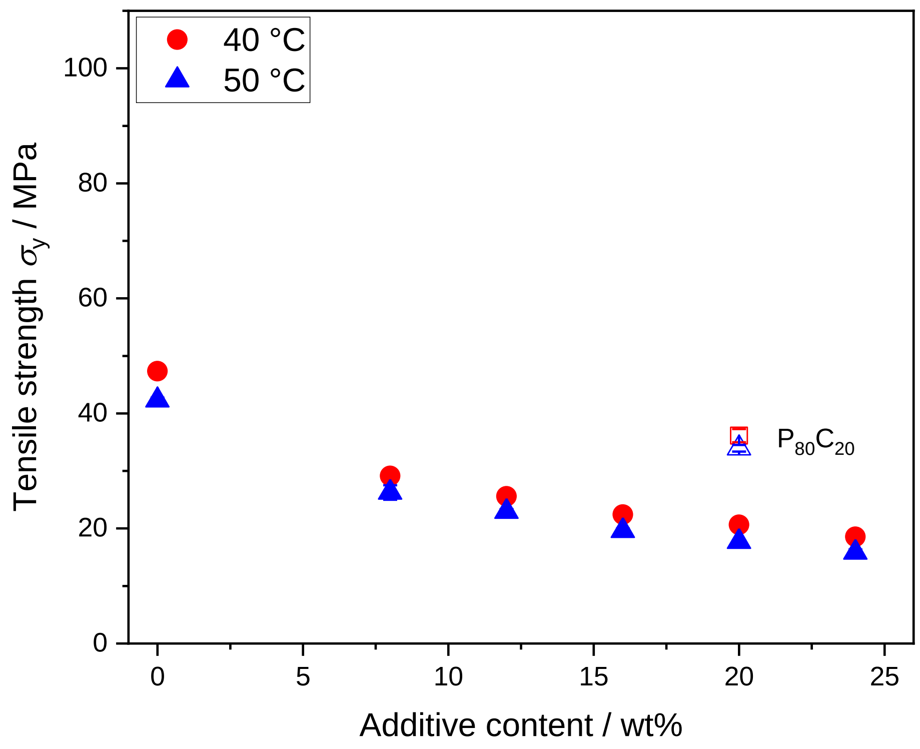

Figure S4: Tensile strength as a function of total additive content at 40 and 50 °C.

Table S2: Thermal properties of the specimens after annealing at 130 °C for 2 h.

Specimen	T_g [°C]	T_m [°C]	ΔH_m [J·g⁻¹]	X_c [%]	w_{PLA} [%]
P_{100}	62.6	152.7	35.53	38%	100
$P_{80}C_{20}$	59.4	153.7	35.84	48%	80
$P_{92}C_6L_2$	61.8	154.8	36.86	43%	92
$P_{88}C_9L_3$	56.5	153.8	37.02	45%	88
$P_{84}C_{12}L_4$	58.7	153.4	35.54	45%	84
$P_{80}C_{15}L_5$	57.2	152.7	33.78	45%	80
$P_{76}C_{18}L_6$	56.7	153.1	32.16	45%	76

Table S3: Tensile properties of the specimens after annealing at 130 °C for 2 h.

Specimen	Young's modulus E [GPa]	Tensile strength σ_y [MPa]	Fracture strain ε_b [%]
P_{100}	4.63 ± 0.87	49.45 ± 3.09	4.13 ± 0.36
$P_{80}C_{20}$	5.93 ± 0.74	23.84 ± 3.25	1.20 ± 0.32
$P_{92}C_6L_2$	4.44 ± 0.92	34.61 ± 1.01	2.98 ± 0.08
$P_{88}C_9L_3$	4.94 ± 0.72	28.15 ± 0.89	1.96 ± 0.14
$P_{84}C_{12}L_4$	4.54 ± 0.44	22.77 ± 1.05	1.66 ± 0.14
$P_{80}C_{15}L_5$	5.22 ± 0.29	16.31 ± 0.38	1.13 ± 0.02
$P_{76}C_{18}L_6$	4.21 ± 0.05	13.41 ± 0.62	1.02 ± 0.16

Table S4: Relative changes in tensile properties after annealing at 130 °C for 2 h. The relative changes were calculated as $\Delta X = (X_{annealed} - X_{as\text{-}moulded}) / X_{as\text{-}moulded} \cdot 100\ \%$.

Specimen	Young's modulus ΔE [%]	Tensile strength $\Delta\sigma_y$ [%]	Fracture strain $\Delta\varepsilon_b$ [%]
P_{100}	389.5 ± 100.4	−20.8 ± 5.0	−39.9 ± 7.4
$P_{80}C_{20}$	370.4 ± 59.4	−52.7 ± 6.5	−67.4 ± 9.2
$P_{92}C_6L_2$	455.1 ± 121.4	−21.6 ± 2.3	−89.1 ± 1.5
$P_{88}C_9L_3$	514.9 ± 91.3	−27.7 ± 2.3	−90.5 ± 2.4
$P_{84}C_{12}L_4$	497.0 ± 59.8	−31.4 ± 3.2	−92.5 ± 1.1
$P_{80}C_{15}L_5$	577.7 ± 42.9	−45.9 ± 1.3	−92.4 ± 1.9
$P_{76}C_{18}L_6$	499.9 ± 12.8	−47.5 ± 2.7	−86.4 ± 3.2
